# East Anglian early Neolithic monument burial linked to contemporary Megaliths

**DOI:** 10.1080/03014460.2019.1623912

**Published:** 2019-06-11

**Authors:** Christiana L. Scheib, Ruoyun Hui, Eugenia D’Atanasio, Anthony Wilder Wohns, Sarah A. Inskip, Alice Rose, Craig Cessford, Tamsin C. O’Connell, John E. Robb, Christopher Evans, Ricky Patten, Toomas Kivisild

**Affiliations:** a Estonian Biocentre, Institute of Genomics, University of Tartu, Tartu, Estonia;; b McDonald Institute for Archaeological Research, University of Cambridge, Cambridge, UK;; c Department of Human Genetics, KU Leuven, Leuven, Belgium;; d Big Data Institute, University of Oxford, Oxford, UK;; e Cambridge Archaeological Unit, Cambridge, UK;; f Department of Archaeology, University of Cambridge, Cambridge, UK

**Keywords:** Ancient DNA, kinship, Britain, isotope analysis

## Abstract

In the fourth millennium BCE a cultural phenomenon of monumental burial structures spread along the Atlantic façade. Megalithic burials have been targeted for aDNA analyses, but a gap remains in East Anglia, where Neolithic structures were generally earthen or timber. An early Neolithic (3762–3648 cal. BCE) burial monument at the site of Trumpington Meadows, Cambridgeshire, UK, contained the partially articulated remains of at least three individuals. To determine whether this monument fits a pattern present in megalithic burials regarding sex bias, kinship, diet and relationship to modern populations, teeth and ribs were analysed for DNA and carbon and nitrogen isotopic values, respectively. Whole ancient genomes were sequenced from two individuals to a mean genomic coverage of 1.6 and 1.2X and genotypes imputed. Results show that they were brothers from a small population genetically and isotopically similar to previously published British Neolithic individuals, with a level of genome-wide homozygosity consistent with a small island population sourced from continental Europe, but bearing no signs of recent inbreeding. The first Neolithic whole genomes from a monumental burial in East Anglia confirm that this region was connected with the larger pattern of Neolithic megaliths in the British Isles and the Atlantic façade.

## Introduction

**Figure 1.**
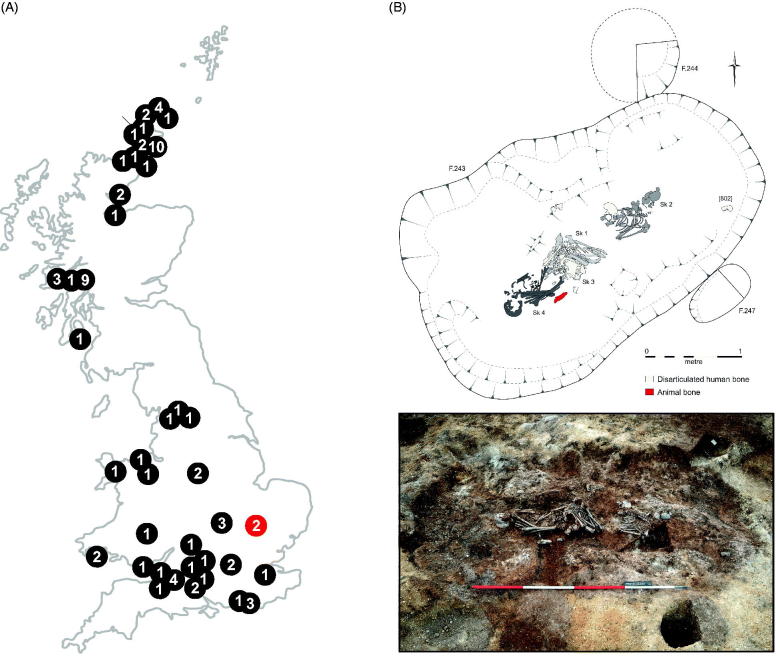
(A) Location and numbers of sequenced individuals from Neolithic contexts in the UK. Trumpington Meadows is marked in red, other published data is black, the number represents the number of genomes from the site. (B) Site layout of burial in Monument I with photograph (modified with permission from Evans et al. (2018)).

Recent archaeogenomic research has mainly focused on the broad genetic affinities of British Mesolithic (*n* = 6), Neolithic (*n* = 83) and Bronze Age (*n* = 67) people and population turnover coinciding with material culture change (Olalde et al. 2018; Brace et al. 2019), with one study focusing on the connections within and between megalithic burials along the Atlantic façade (Sanchez-Quinto et al. 2019). To date, Neolithic sites from the easternmost section of southern Britain (East Anglia) remain uncharacterised (Figure 1A). At the site of Trumpington Meadows, Cambridgeshire, two adjacent, broadly contemporary monuments, erected as early as 3762–3648 cal. BCE and modified through the Early Bronze Age (EBA), were excavated revealing an internment of at least three partially articulated and disarticulated older middle/mature adult males in Monument I (Figure 1B) (Evans et al. 2018). Two of the individuals, Sk.4/799 and Sk.1/880, were buried in close association at the south end of the burial chamber with a third disarticulated Sk.3/800 overlying Sk.1/880 that may be the same individual, while another, Sk.2/801, lay apart from them at the north end. Skeleton 4/799 was associated with a leaf-shaped arrowhead, which could suggest his death through violence (Evans et al. 2018). All individuals were directly radiocarbon dated (Table 1), suggesting usage of the burial chamber over 50–75 years (Evans et al. 2018).

**Table 1.** Archaeological information, mtDNA, Y chromosome, genetic sex and kinship of the samples in the study.

Skeleton	^14^C Date	Sex		Age	MT hg	Y hg	Kinship	Source
			Morph.						Gen.
Sk.4/799	4712 ± 24	nd	XY	Older Middle Adult	K1a + 195	I2d-Y3709 / I2a2a	1st Degree of 880	This study
Sk.1/880	4921 ± 24	Male	XY	Middle/Mature Adult	K1a + 195	I2d-Y3709 / I2a2a	1st Degree of 799	This study
Sk.3/800	4847 ± 24	Male	nd	Mature Adult	nd	nd	nd	Evans et al. (2018)
Sk.2/801	4886 ± 25	Male	nd	Older Middle/Mature Adult	nd	nd	nd	Evans et al. (2018)

All dates are ^14^C dates uncalibrated.

MT hg, mitochondrial DNA haplogroup; Y hg, Y chromosome haplogroup.

## Subjects and methods

Three individuals were sampled from the available skeletal elements with petrous bone preferred if available: Sk.2/801 (petrous bone), Sk.1/880 (tooth) and Sk.4/799 (tooth). Sk.3/800 lacked teeth and petrous bone and is likely the same individual as Sk.1/880, thus was not sampled at this time. Ancient DNA (aDNA) was successfully extracted in a dedicated facility (see Supplementary material) from the teeth while the extract from the petrous bone failed to amplify. Extracts were built into double-stranded, single-indexed libraries and were shotgun-sequenced on Illumina NextSeq500 using the single-end 75 base pair kit. For details on analysis pipelines and software, see Supplementary materials. Carbon and nitrogen isotopic values were measured on bone collagen extracted from the ribs of both Sk.4/799 and Sk.1/880, as well as the individual Sk.2/801, using continuous flow isotope ratio mass spectrometry (Table 2) (see Supplementary material).

**Table 2.** Isotopic information from skeletons of Monument I.

Skeleton	Sample label	Element sampled	δ13C (‰)	δ15N (‰)	%C	%N	C/N ratio
Sk.2/801	TRM10_243_R	Rib	−20.9	10.5	39.6	14.8	3.1
Sk.2/880	TRM10_294_R	Rib	−21.0	10.1	34.2	12.6	3.2
Sk.2/799	TRM10_248_R	Rib	−20.8	10.1	38.7	14.4	3.1

## Results

### Data quality and authenticity

The endogenous human DNA content for Sk.1/880 and Sk.4/799 was 38.4 and 55.8%, respectively, both individuals yielded full mitochondrial (134–140X) and nuclear DNA (1.3–1.9X) including the Y chromosome (0.27–0.40X) (Table S2). Both mitochondrial and nuclear DNA contamination estimates are low (0.40–1.22% and 0.73–0.89%, respectively, Table S2) and both libraries show damage patterns of on average 48–52% C > T deamination in the first 5 bp from the 5’ ends (Table S3). Collagen was successfully extracted from the ribs and analysed. Quality criteria (C/N ratio, %C, %N) show that the collagen was well-preserved (see Supplementary material).

### Relationship of the ancient individuals to each other

The mitochondrial (K1a + 195) (Table S4) and Y chromosome (I2d-Y3709 or I2a2a) (Table S5) haplotypes were identical, indicating both a maternal and paternal relationship. To investigate with higher resolution, we ran READ (Kuhn et al. 2018) on the autosomal data of these two samples together with 68 (Sanchez-Quinto et al. 2019) published Neolithic samples (Olalde et al. 2018) and detected a first degree relationship between the two Trumpington samples (Table S6). To distinguish between a father–son and full-sibling relationship, genotypes were imputed using the 1000 Genomes Project European reference panel (The 1000 Genomes Project Consortium et al. 2015) and genetic relatedness coefficients calculated in PLINK (Chang et al. 2015) (see Supplementary material). Sk.4/799 and Sk.1/880 are identical by descent (IBD) for one haplotype across 48% of their genome and share IBD for both haplotypes across 33.7% of the genome, which is consistent with a full sibling relationship (Table S7). The radiocarbon determinations indicate that Skeleton 1 probably died before Skeleton 4 and, given their relative ages at death, Skeleton 1 was almost certainly the older sibling.

To explore the size of the Trumpington Meadows Neolithic population and to test for inbreeding in the parents or grandparents of the siblings, we estimated the lengths of the runs of homozygosity (ROH) in PLINK (Chang et al. 2015) using parameter settings described previously (Gamba et al. 2014). Total ROH lengths were higher than 150 MB in both brothers, which is relatively high compared to the range of 70–135 MB in Neolithic genomes from Hungary (Gamba et al. 2014) and observed averages of 105–125 MB in present-day European populations (Table S8, Figure S1). Since we do not observe very long ROH tracts, > 10 MB, in either brother, it is more likely that the high levels of their genomic homozygosity can be explained by their small effective population size, rather than by recent inbreeding in their family. Interestingly the brothers differ from each other in that Sk.1/880 carries two medium-sized segments (∼6 MB), while Sk.4/799 carries none longer than 5 MB. Carrying >5 MB segments is not unusual among modern Europeans: within a pool of 503 modern Europeans studied by the 1KGP, 67 (13.3%) had at least one >5 MB ROH segment, with six (6.8%) of the GBR genomes carrying higher total ROH tract lengths than Sk.1/880 (Table S7).

### Relationship of the ancient individuals to other ancient and modern populations

**Figure 2.**
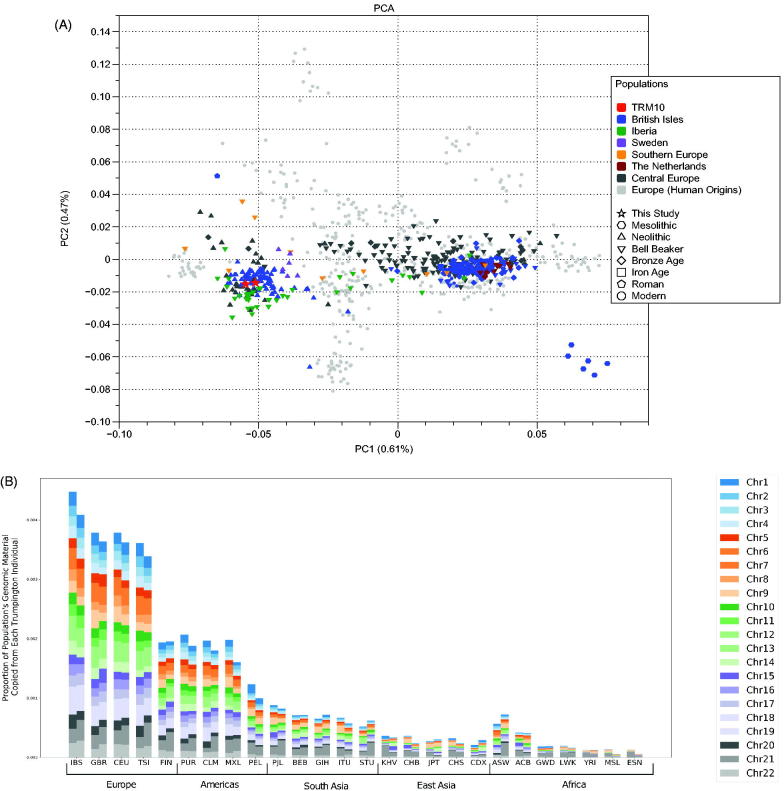
(A) Principal component analysis of ancient samples from Olalde et al. (2018), Brace et al. (2019), and Sanchez-Quinto et al. (2019) projected onto modern variation from 1 KGP (The 1000 Genomes Project Consortium et al. 2015). (B) Genomic descent from the Trumpington samples in the Thousand Genomes Project. Bar height indicates the relative proportion of genomic material in the named population, which is inferred to copy directly from the haplotypes present in Sk.4/799 (left side of each bar) and Sk.1/880 (right side of each bar). Colours give the contribution from each chromosome, relative to chromosome size, to indicate if the pattern differs for different chromosomes. Populations are sorted by super population and decreasing values of the genomic descent statistic (averaged between Sk.4/799 and Sk.1/880). See Supplementary methods for a full description of the genomic descent statistic.

The Y chromosome haplotype I2d-Y3709 or I2a2a (ISOGG) has a modern distribution, mainly in the UK and Ireland (https://www.yfull.com/tree/I-Y3709). All previously published Neolithic British males had I2 haplotypes and three Scottish males had I2d-Y3709 (Karmin et al. 2015; Olalde et al. 2018; Sanchez-Quinto et al. 2019). Haploid genotype calls at 1,233,013 SNPs of the ‘1240 capture’ array (Mathieson et al. 2015) were made in ANGSD (Korneliussen et al. 2014) for both Trumpington individuals (TRM10) and merged with previously published Neolithic British samples (with coverage of >10% SNPs) and modern European genomes from the Human Origins dataset (see Supplementary material), obtaining ∼500k overlapping autosomal SNPs for principal component analysis (PCA). Both individuals cluster together with previously published Neolithic individuals from Britain, Iberia, and Sweden, as well as beaker-associated individuals from Iberia (Olalde et al. 2018) (Figure 2A). F4 statistics in the form of (TestPop1,TestPop2;TRM10,Mbuti.DG) were used to determine any excess allele sharing between TRM10 and other populations. The brothers share excess alleles (*Z* > 3) with Neolithic individuals from the British Isles, France and Iberia when compared to Central European Neolithic individuals, and to Iberian Bell Beaker individuals when compared to English Bronze Age populations (Table S9).

To infer descent from the Trumpington haplotypes within the 1KGP, phased and imputed ancient genomes were made available as potential ancestors of modern samples using *tsinfer*, a newly-developed method for inferring full gene genealogies (Kelleher et al. 2018) (see Supplementary material). We report the relative proportion of the aggregated genetic material of each 1KGP population which ‘copies’ or putatively descends from the Trumpington samples (Figure 2B). Among the 1 KGP super-populations, the greatest amount of genetic material derived from Trumpington haplotypes is found in European populations, followed by admixed American populations. Iberians (IBS) show the greatest amount of genetic material attributable to the Trumpington haplotypes, consistent with recent findings (Olalde et al. 2018).

### Phenotypic and isotopic analysis

Hair, eye and skin colour were predicted using 41 variants in the Hirisplex-S (Chaitanya et al. 2018) (see Supplementary methods). Both brothers have alleles with a predicted phenotype of brown eyes, brown/dark brown hair and intermediate skin; however, they are not identical, with Sk.1/880 predicted to have slightly darker skin pigmentation than his brother (Table S10). Like the majority of the British Neolithic individuals (Olalde et al. 2018), both brothers are homozygous (AA) for the light skin allele (rs1426654 G > A; minor allele A) of the *SLC24A5* gene which is associated with the Anatolian Farmer ancestry (Mathieson et al. 2015); however, they are both heterozygous (GC) for the rs16891982 C > G (minor allele C) SNP in *SLC45A2* which showed a dramatic allele frequency increase from 25% in Neolithic to ∼ 70% in Bronze Age Britain after the arrival of the Steppe Ancestry associated with Bell Beaker culture (Olalde et al. 2018). Like other Neolithic individuals, both brothers lack the derived lactase persistence allele (rs4988235 G > A in *MCM6*; minor allele A). As genetic predisposition and actual diet practices are not intrinsically aligned, carbon and nitrogen isotopic values were measured on the rib bone collagen of the brothers as well as the additional male (Sk. 2/802) interned in the feature to estimate dietary intake. The range of δ^13^C and δ^15^N values is small, with δ^13^C values between −20.8‰ and −21.0‰ and δ^15^N values between 10.1‰ and 10.5‰. Sk.4/799 and Sk.1/880 have identical δ^15^N values. Sk.2/802 has slightly higher δ^15^N values than the brothers, but this is unlikely to be biologically meaningful in this context. These results are consistent with a terrestrial C_3_ diet, with a small contribution of animal protein, but little or no marine protein, although, without contemporary faunal isotopic data, this is harder to quantify. The isotopic similarity of the three individuals indicates that they consumed a similar diet, isotopically and most probably compositionally, particularly regarding access to animal proteins. It is not possible to differentiate between types, e.g. meat, dairy, eggs, from isotopic analysis alone, thus additional proteomics or DNA analysis of calculus could be beneficial. These results currently represent the only isotopic data from Neolithic human skeletons in Cambridgeshire, although δ^13^C and δ^15^N values are broadly similar to several other isotopic studies of Neolithic material from the south of the UK, both in absolute terms and as regards the restricted variation within the group (e.g. Stevens et al. 2012).

## Discussion

The Trumpington monument is an example of a Long Barrow or Chambered Tomb tradition. While further east than the previously described megalithic burials from the British Isles, it fits within a number of previously observed patterns generic for the monumental burial tradition of the fourth millennium BCE. First, bone collagen carbon and nitrogen isotopic values indicate a terrestrial diet rather than exploitation of marine resources found in British Mesolithic hunter-gatherers (Richards et al. 2003) and show that the three interned individuals shared a similar diet. Second, individuals buried within the Megaliths and in the Trumpington Long Barrow monument all share a genetic affinity to Iberian/Atlantic Façade populations, being more distant to the central European Neolithic individuals (Lazaridis et al. 2014; Olalde et al. 2018) and to later Bell Beaker individuals buried within the same site (Olalde et al. 2018). Third, they share phenotypic alleles similar to Anatolian farmers (Lazaridis et al. 2014) and the burials have a higher proportion of males interred with evidence of close kinship ties within and among the monumental burials (Sanchez-Quinto et al. 2019). The ubiquity of the I2d-Y3709/I2a2a Y chromosome haplogroup in Neolithic British Isles individuals, while mtDNA shows more diversity, supports a sex-biased founder effect in the monumental burial tradition. The population from which these brothers came was smaller than previously studied continental European Neolithic populations, but did not carry marks of recent inbreeding. Further analysis of additional individuals from the monument including palaeopathology, aDNA from bones and dental calculus and/or proteomics could inform on remaining questions regarding the genetic relationship among the other interred individuals, dietary exploitation in Neolithic East Anglia and the presence of and manifestations of disease during this time period.

## Supplementary Material

Supplemental Material

## Data Availability

The data that support the findings of this study are openly available in the European Nucleotide Archive at https://www.ebi.ac.uk/ena, reference number PRJEB31305. Previously published data available in the public domain were derived from the European Nucleotide Archive at https://www.ebi.ac.uk/ena.
