# Supplementary material for: East Anglian early Neolithic monument burial linked to contemporary Megaliths
Source: Ann Hum Biol. 2019 Jun 11;46(2):145–9. doi: 10.1080/03014460.2019.1623912 (PMC6816495; doi:10.1080/03014460.2019.1623912)
Supplement: Supplemental Material [file IAHB_A_1623912_SM1111.pdf]

East Anglian Early Neolithic monument burial linked to contemporary Megaliths – Supplementary Materials

S1: Archaeological Background

Craig Cessford

The Trumpington Meadows site located southwest of Cambridge, UK, was excavated in 2010-11 by the Cambridge Archaeological Unit in advance of development ((Evans et al. 2018). Covering 6.1 hectares, archaeological features spanning the Neolithic to the Anglo-Saxon periods were investigated. Two heavily truncated contemporary adjacent Neolithic round barrows were present, the better preserved Monument I had a complex history with at least four burials and four phases of development that continued into the Early Bronze Age. The burials were probably placed within a timber chamber supported by earthen banks. The human remains comprised the skeletons of three adult males (Skeletons 1 [880], 2 [801] and 4 [799]) plus a deposit of partially articulated and disarticulated bone (Skeleton 3 [800]) that may include both a fourth individual and parts of two of the other individuals. The sequence of interment is uncertain, although it has been argued that the most likely sequence from earliest to latest is Skeleton 1, Skeleton 2, Skeleton 3 and Skeleton 4. The radiocarbon determinations as quoted do not allow for the impact of either reservoir effects or bone turnover/remodelling. It is unlikely that either marine or freshwater fish formed a significant dietary component, so the impact of these can probably be discounted. Different skeletal elements were dated for the different individuals (Skeleton 1 tibia, Skeleton 2 femur, Skeleton 3 rib, Skeleton 4 femur/tibia?), so the impact of bone turnover/remodelling would have varied and be greater for the long bones than the ribs. As all the individuals are adults, bone turnover/remodelling may have had an impact of making the radiocarbon determinations perhaps five to twenty years older than the actual time when the individuals died.

Analysis, which does not take into account either reservoir effects or bone turnover/remodelling but is still broadly robust, using Bcal (https://bcal.sheffield.ac.uk; (Buck et al. 1999)) indicates that the period of burial at Monument probably began between 3763-3662 cal BC) and ended between 3626-3484 cal BC (both 68% highest posterior density (HPD). It has been suggested that the burial chamber is unlikely to have been operational for more than 50 to 75 years ((Evans et al. 2018)p. 80), while analysis suggests that it was in use for at least 28 (95% HPD) or 46 (68% HPD) years. Concerning the two individuals with a full-sibling relationship there is a 96.5% probability that Skeleton 1 died before Skeleton 4 and at a 68% HPD Skeleton 1 probably died 28 to 105 years before Skeleton 4, while at the 95% HPD Skeleton 1 probably died between 23 and 293 years before Skeleton 4 (Bcal analysis)

S2: Isotope Data Generation

Alice Rose, Tamsin O'Connell

Preparation of samples for Isotopic analysis

Samples for carbon and nitrogen isotope analysis were prepared using a modified version of the Longin method (Longin 1971). Approximately 500mg of bone was cut using a hand-held

dremel drill with a diamond tipped cutting wheel. The bone surface was then abraded using a precision sandblaster to remove surface dirt and contaminants. Samples were placed into pre-weighed glass test tubes and approximately 8ml of 0.5M aq. HCl was added. The samples were agitated twice a day and 0.5M aq. HCl replaced as appropriate until full demineralisation. When all samples were demineralised, samples were rinsed 3 times with distilled water, then the distilled water was removed and approximately 8ml of pH 3.0 water was added. The samples were then heated in an oven for 48hrs at 75°C until gelatinised. Once partially cooled, the supernatant liquor was filtered using Ezee filters, frozen, then lyophilised.

Analysis was carried out using a Costech automated elemental analyser coupled with a Thermo Finnigan Delta V mass spectrometer in the Godwin Laboratory, Department of Earth Sciences, Cambridge. All samples were run in triplicate (0.8mg ±0.1mg). Data Carbon and nitrogen stable isotope values are expressed as delta values (e.g. $\delta^{13}C$) on the VPDB and AIR scales, for carbon and nitrogen respectively (Coplen 2011).

The ‘quality’ of the collagen was deemed acceptable if the C:N fell within the accepted range of 2.9 – 3.6 (DeNiro 1985). The preservation and purity of the collagen was deemed acceptable if the samples contained more than 1% collagen by mass, with a composition of more than 13%C and 4.8%N (Ambrose 1990). Any results outside of these values were rejected.

S3: Ancient DNA Data Generation

Christiana Scheib

Sampling, decontamination and extraction

Inside a class IIB hood in the dedicated aDNA facility of the University of Tartu Institute of Genomics, root portions of teeth were removed with a sterile drill wheel. Petrous was sampled with a 10mm core drill sterilised with bleach followed by distilled water and then ethanol rinse.

Root and petrous portions were briefly brushed to remove surface dirt, any varnish or lacquer, and microbial film with full strength household bleach (6% w/v NaOCl) using a disposable toothbrush that was soaked in 6% (w/v) bleach prior to use. They were then soaked in 6% (w/v) bleach for 5 minutes. Samples were rinsed twice with 18.2 MΩcm H_2O and soaked in 70% (v/v) Ethanol for 2 minutes, transferred to a clean paper towel on a rack inside a class IIB hood with the UV light on and allowed to dry. They were weighed and transferred to PCR-clean 5 ml or 15 ml conical tubes (Eppendorf) for chemical extraction.

Inside a class IIB hood, per 100 mg of each sample, 2 ml of 0.5M EDTA Buffer pH 8.0 (Fluka) and 50 µl of Proteinase K 10 mg/ml (Roche) was added. Tubes were rocked in an incubator for 72 hours at room temperature. Extracts were concentrated to 250 µl using Amplicon Ultra-15 concentrators with a 30 kDa filter (Millipore).

Samples were purified according to manufacturer’s instructions using buffers from the Minelute^{TM} PCR Purification Kit (Qiagen) with the following changes: 1) the use of High-Volume spin columns (Roche); 2) 10X PB buffer instead of 5X; and 3) samples incubated

with EB buffer (Qiagen) at 37C for 10 minutes prior to elution. The columns were transferred to clean, labelled, 1.5ml Eppendorf tubes. One hundred microlitres EB buffer is added to the membrane and centrifuged at 13,000 rpm for two minutes after the 10-minute incubation and stored at -20 C. Only one extraction was performed per sample for screening and 30μl used for libraries.

Library amplification

Library preparation was conducted using a protocol modified from the manufacturer's instructions included in the NEBNext® Library Preparation Kit for 454 (E6070S, New England Biolabs, Ipswich, MA) as detailed in (Meyer & Kircher 2010). DNA was not fragmented and reactions were scaled to half volume, adaptors were made as described in (Meyer & Kircher 2010) and used in a final concentration of 2.5uM each. DNA was purified on MinElute columns (Qiagen). Libraries were amplified using the following PCR set up: 50μl DNA library, 1X PCR buffer, 2.5mM MgCl2, 1 mg/ml BSA, 0.2μM inPE1.0, 0.2mM dNTP each, 0.1U/μl HGS Taq Diamond and 0.2μM indexing primer. Cycling conditions were: 5' at 94C, followed by 18 cycles of 30 seconds each at 94C, 60C, and 68C, with a final extension of 7 minutes at 72C. Amplified products were purified using MinElute columns and eluted in 35 μl EB (Qiagen). Three verification steps were implemented to make sure library preparation was successful and to measure the concentration of dsDNA/sequencing libraries – fluorometric quantitation (Qubit, Thermo Fisher Scientific), parallel capillary electrophoresis (Fragment Analyser, Advanced Analytical) and qPCR.

S4: Ancient DNA Data Analysis

Christiana Scheib, Ruoyun Hui, Toomas Kivisild, Eugenia D'Atanasio

Mapping and Genotyping

The samples were shotgun-sequenced on the Illumina NextSeq500 using single-end 75 base pair kit. Before mapping, the sequences of adaptors and indexes and poly-G tails occurring due to the specifics of the NextSeq 500 technology were removed from the ends of DNA sequences using cutadapt 1.9 (Martin 2011). Sequences shorter than 30 bp were also removed with the same program to avoid random mapping of sequences from other species.

The sequence reads were mapped to reference sequence GRCh37 (hg19) using Burrows-Wheeler Aligner (BWA 0.7.12) (Li & Durbin 2009) command `aln` with seeding disabled.

After mapping, the sequences were converted to BAM format and only sequences that mapped to the human genome were kept with samtools 1.9 (Li et al. 2009). Next, multiple bams from the same individual, but different runs were merged using samtools merge, reads with mapping quality under 30 were filtered out and duplicates were removed with picard 2.12 (http://broadinstitute.github.io/picard/index.html).

aDNA Authentication

As a result of degradation over time, aDNA can be distinguished from modern DNA by certain characteristics: short fragments and a high frequency of C > T substitutions at the 5'

ends of sequences due to cytosine deamination. The program mapDamage2.0 (Johnsson et al. 2013) was used to estimate the frequency of 5' C > T transitions.

mtDNA contamination was estimated using the method from (Jones et al. 2017), which aligns the raw mtDNA reads to the RSRS (Behar et al. 2012), determines the haplotype using GATK pileup (McKenna et al. 2010) counts the number of heterozygous reads on haplotype-defining sites as well as adjacent sites and calculates a ratio that takes into account ancient DNA damage by excluding positions where a major allele is C or G and the minor is T or A respectively.

On average, the samples showed 48% - 52% C > T substitutions in the first five base pairs of the 5' ends (Table S7). The mtDNA contamination estimate for samples ranged from 0.4% to 1.22% with an average of 0.92% (Table S2). The average of the two X chromosome contamination methods was between 0.19 and 2.27% with an average of 0.71% (Table S2).

Calculating general statistics and determining genetic sex

Samtools-1.9 (Li et al. 2009) option stats was used to determine the number of final reads, average read length, average coverage etc.

Genetic sex was calculated using the freely available python script from (Skoglund et al. 2013), which estimates the fraction of reads with mapping quality > 30 mapping to Y chromosome out of all reads mapping to either X or Y chromosome. Genetic sexing confirmed morphological sex estimates for both individuals.

Variant calling

Variants were called in two ways, 1) for PCA and F statistics, pseudo-haploid genotypes were called with ANGSD (Korneliussen et al. 2014) command –doHaploCall 1, sampling a random base for the positions that are present in the 1.24 million SNPs present in comparative datasets; 2) for imputation, genotype likelihoods of 2.8 million SNPs with a minor allele count >= 5 in the 1000 Genomes Project Phase 3 (1KG) were called using the ATLAS pipeline (Link et al. 2017), in which the base quality was recalibrated according to post-mortem damage pattern estimated from heterozygous reads on chromosome X.

Global Imputation

The genotypes were first estimated with BEAGLE 4.1(Browning & Browning 2007) from genotype likelihoods produced by ATLAS (Link et al. 2017) in Beagle -gl mode, followed by imputation in Beagle -gt mode with BEAGLE 5 (Browing et al 2018) from sites where the GP of the most likely genotype reaches 0.99. To balance between imputation times and accuracy, we used 503 Europeans genomes in 1KG as the reference panel in Beagle -gl step, and 27,165 genomes (except for chromosome 1, where the sample size is reduced to 22,691 due to a processing issue in the release) from the Haplotype Reference Consortium (HRC) (The Haplotype Reference Consortium, 2016) in the Beagle -gt step.A second GP filter (MAX(GP) >= 0.99) was applied after the imputation. The imputation accuracy evaluated by running the same pipeline after down-sampling the sequencing reads of chromosome 20 of a Neolithic Hungarian genome (Gamba et al. 2014) to ~1.27X is shown in Table S11. For downstream

analyses using the imputed genome (runs of homozygosity, IBD segments, and tsinfer), we also filtered out variants with a minor allele frequency below 0.3 in the HRC panel.

Determining mtDNA haplogroups

Raw reads were mapped to the revised Cambridge Reference Sequence (rCRS) (Andrews et al. 1999). Variants were called using Samtools 1.9 `mpileup` variant-only option (Li et al. 2009) and filtered using bcftools v 1.1 (Li et al. 2009). Haplogroups were assigned using Phylotree build 16 (van Oven & Kayser 2009) accessed at www.phylotree.org and Haplogrep (Kloss-Brandstatter et al. 2011) accessed at https://haplogrep.uibk.ac.at.

Y chromosome variant calling and haplotyping

Y chromosome variants were called as haploid and picking one allele at random (`--doHaploCall 1`) in ANGSD-0.916 (Korneliussen et al. 2014) and filtered for regions that uniquely map to the Y chromosome, retaining 8.8 Mb when using short read sequencing technology (Karmin et al. 2015). Haplogroup assignments were made on the basis of in silico genotyping of the samples for 108,000 informative variants 1000 Genome Project populations (Poznik et al. 2016) in 456 geographically diverse high-coverage Y chromosome sequences (Karmin et al. 2015) and those annotated by https://isogg.org/tree/ and https://www.yfull.com/. In haplogroup labelling we followed the nomenclature of Karmin et al. (2015) but also included the most recent isogg nomenclature when comparing to recent published work.

Principal component analysis

Following pseudo-haploid genotype calling, we performed principal component analysis (PCA) on the merged dataset containing the Trumpington individuals, previously published ancient genomes from western Europe (Martiniano et al. 2016; Schiffels et al. 2016; Olalde et al. 2018; Brace et al. 2019, Sanchez-Quinto et al. 2019), and modern European genomes from the public Human Origins dataset (Lazaridis 2016) using EIGENSOFT smartpca (Patterson et al. 2006). 504,263 autosomal biallelic SNPs remained after the merge, and ancient samples were projected onto coordinates established by the Human Origins samples.

F statistics

We merged the pseudo-haploid calls of the two Trumpington genomes and other newly published Neolithic British genomes (Brace et al 2019, Sanchez-Quinto et al 2019) with a compiled 1240k capture dataset of ancient and present-day individuals (v37.2) downloaded from https://reich.hms.harvard.edu/downloadable-genotypes-present-day-and-ancient-dna-data-compiled-published-papers in the EIGENSTRAT format. The haploid data were pseudo-diploidized and converted to plink format using ANGSD and merged in Plink-1.9 (Chang et al. 2015) with the available ancient data then converted to EIGENSTRAT format using EIGENSOFT-7.2.0 (Price et al. 2006; Patterson et al. 2006). F4 statistics were calculated using AdmixTools-5.1 (Patterson et al. 2012) with “Mbuti.DG” population as the outgroup.

Kinship analyses

We searched for potential kinship between the two Trumpington individuals and 68 British Neolithic individuals whose genomes have been published. As values are affected by the reference population used, we restricted analysis to only Early Neolithic samples from the

British Isles, excluding samples whose 95% confidence interval of C14 date falls more recent than 4,500 BP (Olalde et al 2018, Brace et al 2019, Sanchez-Quinto et al. 2019). Pseudo-haploid calls at a total of 1,233,013 SNPs of the '1240k capture' produced in ANGSD were converted to .tped format which was used as an input for kinship analyses with READ (Kuhn et al. 2018). Merged bam files were used in the case of multiple runs per sample. Results are reported for relationships between the TRM10 samples and the comparative individuals in Table S6.

The lengths of runs of homozygosity were estimated in PLINK (Chang et al. 2015) using parameter settings described previously (Gamba et al. 2014): --homozyg --homozyg-window-snp 50 --homozyg-snp 50 --homozyg-kb 1600 --homozyg-window-het 1 --homozyg-window-threshold 0.05 --homozyg-gap 100 --homozyg-density 50. The proportion of genome identical by descent was calculated in PLINK using the --genome function. The results of these analyses are reported in Tables S8 and S7, respectively.

Phenotype Prediction

Eugenia D'Atanasio

To predict eye, hair and skin colour of the two Neolithic individuals, we selected all 41 variants from 19 genes in 9 autosomes in the HIrisPlex-S system (Chaitanya et al. 2018). We analysed a total of 10 regions ranging from 1.5 Mb to 6 Mb, obtained by adding 1 Mb at each side of each autosomal segment delimited by HIrisPlex-S variants. The three genes on chromosome 15 have been analysed in two different regions (OCA2-HERC2 region and SLC24A5 region) because the distance between the two nearest SNPs of the two segments was greater than 20 Mb. For each of the 10 regions, we extracted the variants present in a modern reference panel composed of all the samples in the 1000 Genomes Phase 3 (1000 Genomes Project Consortium et al. 2015). We extracted only the biallelic variant sites with a minor allele frequency (MAF) above 0.1%, using VCFtools and bcftools (Li 2012). Using this approach, we excluded only two variants (one SNP and one indel) of the HIrisPlex-S set. The final list of variant sites was obtained by manipulating the VCFs with PLINK 1.9 (Chang et al. 2015). The local imputation was performed using the same pipeline for the global imputation, with some differences. All 2,504 genomes from the 1000 Genomes Project have been used as reference panel at the first Beagle step (-gl), while the HRC reference panel (> 30,000 samples from worldwide cohorts, including the 1000 Genomes Project) was chosen for the Beagle -gt step. After imputation we applied a second GP filter, setting 0.85 as the threshold (Gamba et al. 2014).

The phenotype prediction was performed using the HIrisPlex-S webtool (https://hirisplex.erasmusmc.nl/) (Walsh et al. 2014; Walsh et al. 2017), recoding and formatting the genotype information using PLINK 1.9 and R (R Core Team 2018). The missing genotypes were coded as "NA".

The same pipeline was used to extract the allele information for other five variants: one SNP responsible for the lactase persistence in the European populations (rs4988235 in *MCM6*), three SNPs involved in the protection against leprosy (rs5743618 and rs4833095 in *TLR1* and

rs3135388 in *HLA*) and one 32bp deletion responsible of the HIV resistance (rs333 in *CCR5*). Since indels are not present in the HRC reference panel, we used the 1000 Genomes samples in the Beagle -gt step to impute the rs333 variant.

Concordance between global and local imputation is listed in Table S12.

Modern Descendants Analysis

Anthony Wilder Wohns

The program *tsinfer* seeks to infer sequences of trees representing the complete gene genealogy of a sample using a two-step process: first, the ancestral haplotypes which contributed material to the samples are estimated using heuristic methods. Second, a Li and Stephens copying process is employed to show how sampled genomes copy genetic material from ancestral haplotypes. The analysis conducted in this work augments tree sequences generated from the 1000 Genomes Project (1KGP) (1000 Genomes Project Consortium et al. 2015) and Simons Genome Diversity Panel (SGDP) (Mallick et al. 2016) datasets with the phased and imputed genomes of the two Trumpington individuals. The four chromosomes of the Trumpington individuals are inserted at the lowest level of the copying process, allowing samples in the 1KGP and SGDP to directly copy from the ancient individuals. See (Kelleher et al. 2018) for a full description of *tsinfer* and the copying process.

To augment the previously built tree sequences, it was necessary to find the overlap between the ancient chromosomes and modern genetic material. The imputed ancient samples included 64.2% of the 1KGP sites, leaving an overlap of 24,570,629 sites for analysis. In the SGDP dataset 81.4% of sites overlapped, leaving 12,374,480 sites for analysis. After the non-overlapping sites were pruned from the dataset, we augmented the ancestral tree sequence of each 1KGP and SGDP chromosome with the ancient samples and then matched the modern samples to the ancestral tree sequences.

It is important to note that this copying process does not necessarily mean that the Trumpington individuals were direct genetic ancestors of the modern samples. Instead, it indicates that modern samples descend from individuals genetically indistinguishable from the Trumpington individuals over some parts of their genome.

The resulting tree sequences contain considerable detail about the ancestry of the ancient and modern samples at every site included in the analysis. To gain a high-level understanding of the genomic legacy of the Trumpington individuals we computed the genomic descent statistic, described in the next section.

The code implementing this analysis can be found at https://github.com/awohns/neolithic-tsinfer.

Genomic Descent Statistic:

We define the genomic descent statistic quantifying the relative proportion of genetic material in each population which copies from an ancestral node *u*.

Let *R* be a list of *k* sets of nodes. In our application, R is a list of the populations in the 1KGP or SGDP, with set R_k containing the samples in population *k*. For node *i* in reference set R_k, define $A_{i,u}^{c}$ as the total span of node *i* which copies from node *u* on chromosome *c*. The statistic is averaged over the portion of genome where the node is ancestral to *any* sample from the sets in R, A_u^c. Finally, it is normalized by the size of reference set, $|R_k|$. Thus, the genomic descent statistic of node *u* in reference set *k* on chromosome *c* is:

$$D_{c,R_k}(u) = \frac{\sum_{i \in R_k} A_{i,u}^{c}}{A_u^c * |R_k|} \text{ (Equation 1)}$$

To combine information from the autosomes for a given population, the total genomic descent statistic is calculated by adjusting the value of Equation 1 for each chromosome by the ratio of the size of each chromosome, L_c, to the total genome length, L_g:

$$D_{g,R_k}(u) = \frac{\sum_{c=1}^{22} L_c D_{c,R_k}(u)}{L_g}. \text{ (Equation 2)}$$

The height of the stacked bars in Figure 2B reflects the total genomic descent statistic for each population (the value of Equation 2). The relative size of each coloured section of the stacked bars in Figure 2B reflects the value of Equation 1. Thus, while the total height represents the genomic inheritance from the Trumpington haplotypes, the relative size of coloured regions within the bar shows the contribution from each chromosome *relative to the length of that chromosome*. In other words, long chromosomes such as chromosome 1 will not necessarily show a greater relative contribution than smaller chromosomes. This has been done to highlight any differences between chromosomes in terms of their proportion of genomic descent from Trumpington haplotypes. The evenness of the distribution of colours in the different bars indicates that most chromosomes support the same pattern of genomic descent.

Large deviations between chromosomes, for example in chromosome 22 in the SGDP Itelman population, may be due to phasing or imputation errors in ancient or SGDP samples (see Kelleher et al. 2018 for evidence of phasing errors in some 1KGP and SGDP samples). Variance due to small reference set size (for instance, only two Itelman individuals are included in the SGDP) or small chromosome size may also be potential explanatory factors.

References

1000 Genomes Project Consortium et al. 2015. A global reference for human genetic variation. Nature. 526:68–74.

Ambrose SH. 1990. Preparation and characterization of bone and tooth collagen for isotopic analysis. Journal of Archaeological Science. 17:431–451.

Andrews RM, Kubacka I, Chinnery PF, Lightowiers RN, Turnbull DM, Howell N. 1999. Reanalysis and revision of the Cambridge reference sequence for human mitochondrial DNA. Nat Genet. 23:147.

Behar DM, van Oven M, Rosset S, Metspalu M, Loogväli E-L, Silva NM, Kivisild T, Torroni A, Villems R. 2012. A ``Copernican“” Reassessment of the Human Mitochondrial DNA Tree from its Root. The American Journal of Human Genetics. 90:675–684.

Browning SR, Browning BL. 2007. Rapid and accurate haplotype phasing and missing-data inference for whole-genome association studies by use of localized haplotype clustering. American Journal of Human Genetics. 81:1084–1097.

Buck C, Christen A, James GN. 1999. BCal: an on-line Bayesian radiocarbon calibration tool. Internet Archaeology. 65:808.

Chaitanya L, Breslin K, Zuñiga S, Wirken L, Pośpiech E, Kukla-Bartoszek M, Sijen T, Knijff P de, Liu F, Branicki W, et al. 2018. The HIrisPlex-S system for eye, hair and skin colour prediction from DNA: Introduction and forensic developmental validation. Forensic Science International: Genetics. 35:123–135.

Chang CC, Chow CC, Tellier LC, Vattikuti S, Purcell SM, Lee JJ. 2015. Second-generation PLINK: rising to the challenge of larger and richer datasets. Gigascience. 4:7.

Coplen TB. 2011. Guidelines and recommended terms for expression of stable-isotope-ratio and gas-ratio measurement results. Rapid Communications in Mass Spectrometry. 25:2538–2560.

DeNiro MJ. 1985. Postmortem Preservation and Alteration of Invivo Bone-Collagen Isotope Ratios in Relation to Paleodietary Reconstruction. Nature. 317:806–809.

Evans C, Lucy S, Patten R. 2018. Riversides: Neolithic Barrows, a Beaker Grave, Iron Age and Anglo-Saxon Burials and Settlement at Trumpington, Cambridge. Cambridge: McDonald Institute for Archaeological Research.

Gamba C, Jones ER, Teasdale MD, McLaughlin RL, Gonzalez-Fortes G, Mattiangeli V, czki LASODO, aacute ri von IKO, Pap IO, Anders A, et al. 2014. Genome flux and stasis in a five millenium transect of European prehistory. Nature Communications. 5:1–9.

Johnsson H, Ginolhac A, Shubert M, Johnson PL, Orlando L. 2013. mapDamage2.0: fast approximate Bayesian estimates of ancient DNA damage parameters. Bioinformatics.

29:1682–1684.

Jones ER, Zariņa G, Moiseyev V, Lightfoot E, Nigst PR, Manica A, Pinhasi R, Bradley DG. 2017. The Neolithic Transition in the Baltic Was Not Driven by Admixture with Early European Farmers. Curr Biol. 27:576–582.

Karmin M, Saag L, Vicente M, Sayres MAW, Jarve M, Talas UG, Rootsi S, Ilumaee A-M, Maegi R, Mitt M, et al. 2015. A recent bottleneck of Y chromosome diversity coincides with a global change in culture. Genome Research. 25:459–466.

Kelleher J, Wong Y, Albers PK, Wohns AW, McVean G. 2018. Inferring the ancestry of everyone. bioRxiv.:458067.

Kloss-Brandstatter A, Pacher D, Schonherr S, Weissensteiner H, Binna R, Specht G, Kronenberg F. 2011. HaploGrep: a fast and reliable algorithm for automatic classification of mitochondrial DNA haplogroups. Hum Mutat. 32:25–32.

Korneliussen TS, Albrechtsen A, Nielsen R. 2014. ANGSD: Analysis of Next Generation Sequencing Data. BMC Bioinformatics. 15:356.

Kuhn JMM, Jakobsson M, Gunther T. 2018. Estimating genetic kin relationships in prehistoric populations. PLoS ONE. 13:e0195491.

Li H, Durbin R. 2009. Fast and accurate short read alignment with Burrows-Wheeler transform. Bioinformatics. 25:1754–1760.

Li H, Handsaker B, Wysoker A, Fennell T, Ruan J, Homer N, Marth G, Abecasis G, Durbin R, 1000 Genome Project Data Processing Subgroup. 2009. The Sequence Alignment/Map format and SAMtools. Bioinformatics. 25:2078–2079.

Li H. 2012. A statistical framework for SNP calling, mutation discovery, association mapping and population genetical parameter estimation from sequencing data. arXiv. q-bio.GN:2987–2993.

Link V, Kousathanas A, Veeramah K, Sell C, Scheu A, Wegmann D. 2017. ATLAS: Analysis Tools for Low-depth and Ancient Samples. bioRxiv.:105346.

Longin R. 1971. New method of collagen extraction for radiocarbon dating. Nature. 230:241–242.

Mallick S, Li H, Lipson M, Mathieson I, Gymrek M, Racimo F, Zhao M, Chennagiri N, Nordenfelt S, Tandon A, et al. 2016. The Simons Genome Diversity Project: 300 genomes from 142 diverse populations. Nature. 538:201–.

Martin M. 2011. Cutadapt removes adapter sequences from high-throughput sequencing reads. EMBnetjournal. 17:10–12.

McKenna A, Hanna M, Banks E, Sivachenko A, Cibulskis K, Kernytsky A, Garimella K, Altshuler D, Gabriel S, Daly M, DePristo MA. 2010. The Genome Analysis Toolkit: a MapReduce framework for analyzing next-generation DNA sequencing data. Genome

Research. 20:1297–1303.

Meyer M, Kircher M. 2010. Illumina Sequencing Library Preparation for Highly Multiplexed Target Capture and Sequencing. Cold Spring Harbor Protocols. 2010:pdb.prot5448–pdb.prot5448.

Patterson N, Moorjani P, Luo Y, Mallick S, Rohland N, Zhan Y, Genschoreck T, Webster T, Reich D. 2012. Ancient Admixture in Human History. Genetics. 192:1065–.

Patterson N, Price AL, Reich D. 2006. Population structure and eigenanalysis. PLoS Genet. 2:2074–2093.

Poznik GD, Xue Y, Mendez FL, Willems TF, Massaia A, Wilson Sayres MA, Ayub Q, McCarthy SA, Narechania A, Kashin S, et al. 2016. Punctuated bursts in human male demography inferred from 1,244 worldwide Y-chromosome sequences. Nat Genet. 48:593–599.

Price AL, Patterson NJ, Plenge RM, Weinblatt ME, Shadick NA, Reich D. 2006. Principal components analysis corrects for stratification in genome-wide association studies. Nat Genet. 38:904–909.

R Core Team. 2018. R: A language and environment for statistical computing. Vienna, Austria. http://www.R-project.org.

Skoglund P, Storå J, Götherström A, Jakobsson M. 2013. Accurate sex identification of ancient human remains using DNA shotgun sequencing. Journal of Archaeological Science. 40:4477–4482.

van Oven M, Kayser M. 2009. Updated comprehensive phylogenetic tree of global human mitochondrial DNA variation. Hum Mutat. 30:E386–E394.

Walsh S, Chaitanya L, Breslin K, Muralidharan C, Bronikowska A, Pośpiech E, Koller J, Kovatsi L, Wollstein A, Branicki W, et al. 2017. Global skin colour prediction from DNA. Hum Genet. 136:847–863.

Walsh S, Chaitanya L, Clarisse L, Wirken L, Draus-Barini J, Kovatsi L, Maeda H, Ishikawa T, Sijen T, de Knijff P, et al. 2014. Developmental validation of the HIrisPlex system: DNA-based eye and hair colour prediction for forensic and anthropological usage. Forensic Science International: Genetics. 9:150–161.

East Anglian Early Neolithic monument burial linked to contemporary Megaliths – Supplementary Materials

Figure S1.

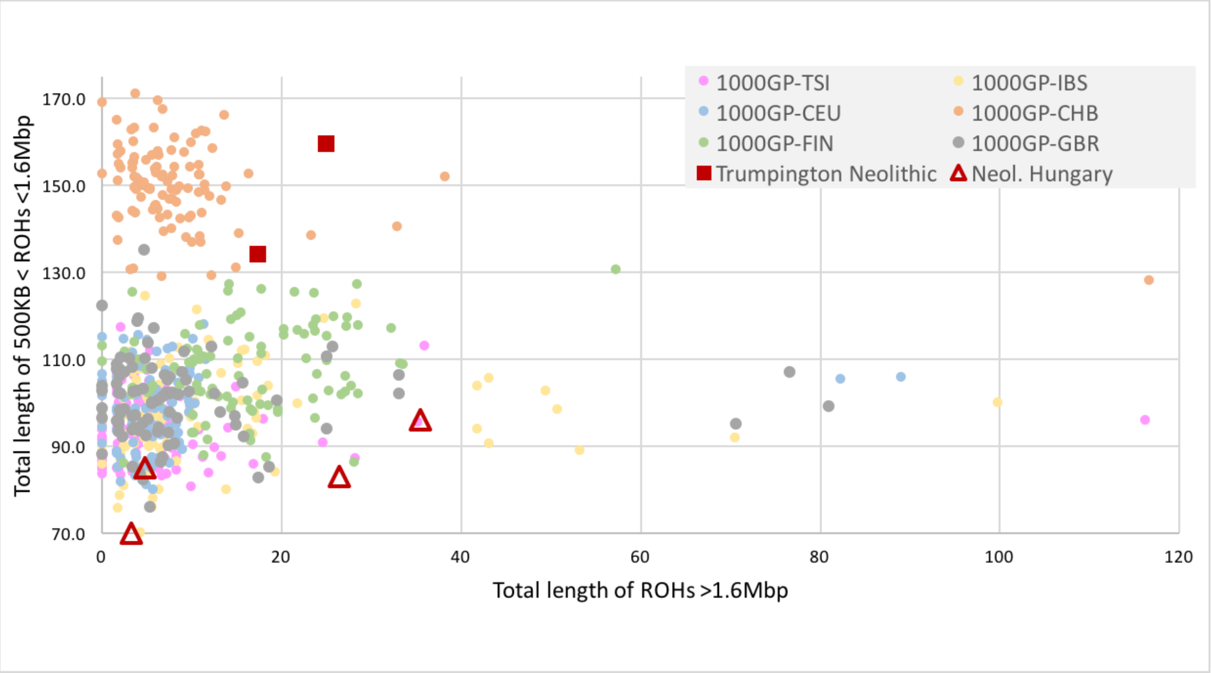

Figure S2.

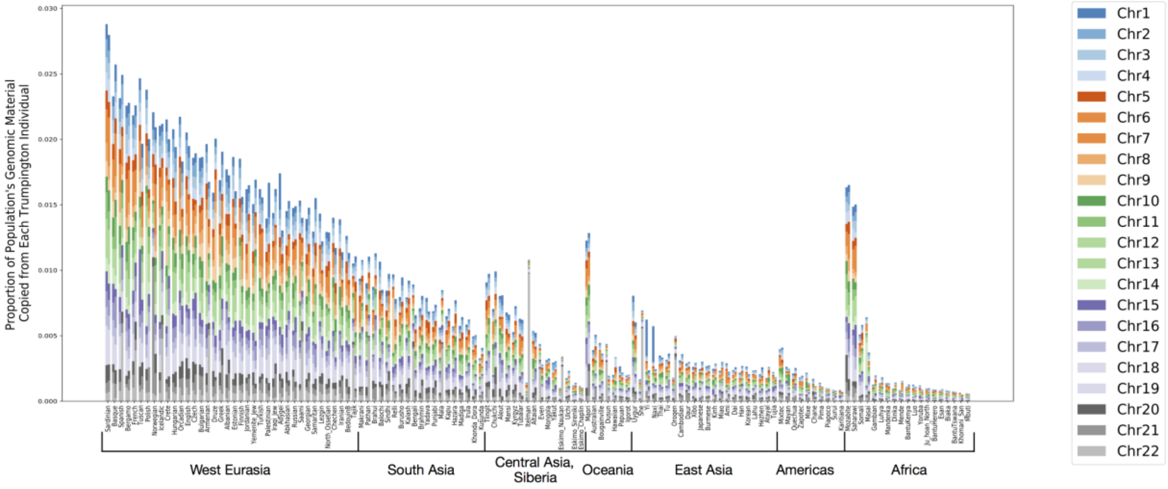

Figure Captions:

1. **Short vs. long runs of homozygosity (ROHs) in Neolithic and Present-day samples.** The lengths of runs of homozygosity estimated in PLINK (Chang et al. 2015) using parameter settings described previously (Gamba et al. 2014). X axis is the number of total lengths of ROH over 1.6 megabase pairs, Y axis is the number of total lengths

over 500 kilobase pairs but less than 1.6 megabase pairs. Ancient samples are plotted against comparative samples from the 1000 Genomes Project (1000 Genomes Project Consortium et al. 2015).

2. **Genomic descent from the Trumpington samples in the Simons Genome Diversity Panel.** Bar height indicates the relative proportion of genomic material in the named population which is inferred to copy directly from the haplotypes present in Sk.4/799 (left side of each bar) and Sk.1/880 (right side of each bar). Colours give the contribution from each chromosome, relative to chromosome size, to indicate if the pattern differs for different chromosomes. Populations are sorted by super population and decreasing values of the genomic descent statistic (averaged between Sk.4/799 and Sk.1/880).
